# ZnO Nanowires for Feedback-Assisted Tuning of Electromechanical Resonators

**DOI:** 10.1021/acsanm.2c03963

**Published:** 2022-09-28

**Authors:** Andrea Orsini, Christian Falconi

**Affiliations:** † Department of Electronic Engineering, University of Rome Tor Vergata, Via del Politecnico 1, 00133 Rome, Italy; ‡ Facoltà di Ingegneria, Università degli Studi Niccolò Cusano, Via Don Carlo Gnocchi 3, 00166 Rome, Italy

**Keywords:** ZnO nanowires, ZnO nanodampers, ZnO nanowires on quartz microbalances, nanowire-tuning, feedback solution-growth, electromechanical resonators, oscillators, feedback-assisted tuning of electromechanical resonators

## Abstract

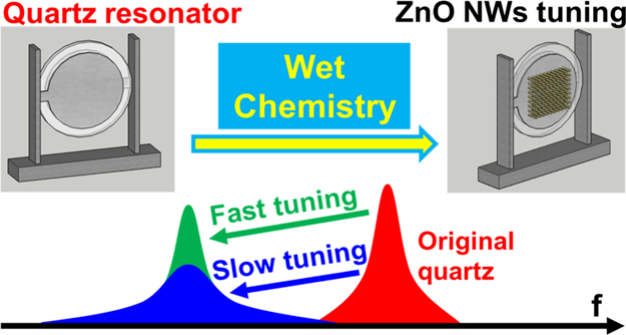

The fabrication of devices with accurately controlled properties almost invariably takes advantage of feedback so that, based on real-time measurements, process parameters can be automatically adjusted in order to obtain the desired characteristics. Nevertheless, despite the outstanding advantages of wet-chemistry methods (e.g., simplicity, low-cost, low-temperature, and compatibility with almost any process and type of substrate), the use of feedback in the solution growth of nanostructures is almost unexplored. In fact, conventional techniques for the real-time in-liquid characterization of nanostructures are extremely complex and can introduce intricate artefacts. Here, by taking advantage of an electro-mechanical resonator as a substrate, we on-line monitor, at the system level, the nanostructure growth, thus enabling the feedback-assisted tuning of low-cost electro-mechanical resonators by ZnO nanowires. This approach allows for post-fabrication tuning of the resonant frequency with high accuracy and high tuning range (e.g., about 1% in our experiments) in a simple, fast, low power, and low-cost manner, without requiring expensive facilities such as clean rooms or high-vacuum deposition systems. Moreover, remarkably, we find that for a given desired resonant frequency, the quality factor of the resonance can be separately adjusted by modifying the nutrient solution, which can be a key advantage for filters. The straightforward interfacing and packaging of the final resonator stems from the large difference, about 5 orders of magnitude, between the key structure dimensions, namely, the diameter of the ZnO nanowires and the much larger (e.g., few millimeters) diameter of the quartz. Our results can lead to the widespread application of nanowire-tuned electro-mechanical oscillators and filters in electronics, sensors, and material science.

## Introduction

Feedback^1^ is a powerful approach for fabricating objects with accurately controlled properties and is, in fact, widely used for micro- and nano-fabrication, including, to name a few, deposition of thin films with controlled thickness,^2^ ion-beam current measurement for controlling the total dose in ion implantation,^2,3^ and feedback-controlled electromigration.^4^ However, the application of feedback to solution-based methods for growing nanostructures^5,6^ is still an open challenge. In fact, on one hand, solution-based growth methods for nanofabrication can have several crucial advantages, including low-cost, large-area, low-temperature deposition on almost arbitrary substrates (including flexible substrates), and compatibility with practically all integrated-circuits and/or MEMS processes; on the other hand, the properties of solution-grown nanostructures are typically affected by large spread and poor reproducibility.^5,7,8^ Nevertheless, feedback control of wet-chemistry nanofabrication processes is almost unexplored because of the extreme complexity of real-time monitoring of the growth of nanostructures in liquids, which would be required for “closing the loop”.^9^ In fact, the available methods (e.g., liquid-cell TEM^10−14^ or X-ray techniques^7,15−18^) are very complex, expensive, add hard-to-estimate artefacts (e.g., heating and formation of bubbles and highly reactive radicals), and are impossible to apply at large scale on standard glass reaction containers (see^19^ for a detailed discussion). Since piezoelectric quartz crystal microbalances (QCMs) can be used as very sensitive mass sensors^20,21^ because of the strong dependence of their resonance frequencies on the mechanical loading on the electrodes, we recently used quartz substrates to on-line monitor the solution-growth of zinc oxide nanowires.^19^ As a proof of concept, we have monitored the growth of ZnO nanorods because of their ease of fabrication^5^ in solution and of the potential of ZnO nanostructures^5,22,23^ for energy, electronics, and sensing. Although different nanostructures could be grown on the electrodes of electro-mechanical resonators for tuning their impedance, ZnO nanowires can be easily grown on almost any substrate.^5,6^ Moreover, a very simple galvanic-assisted procedure for growing ZnO nanowires on the silver electrodes of quartz resonators has also been demonstrated.^19,24^ For these reasons, we have tuned the resonance properties of quartz resonators by simply growing in solution ZnO nanowires on the electrodes of the resonators. Specifically, we adopted a galvanic-assisted, single-step, seedless method for growing high-density, vertically aligned ZnO nanowires on quartz resonators by using the most typical nutrient solution, that is, deionized (DI) water containing an equimolar concentration of zinc nitrate and hexamine (HMTA).^19,24^ Besides, we also demonstrated that the real-time monitoring of the nanowire growth can be used for synthesizing better nanowires in terms of length, aspect ratio, and total deposited ZnO mass.^19^ Here, we grow ZnO nanowires on a quartz electro-mechanical resonator in order to tune its resonant frequency and quality factor, which are key figures of merit for both oscillators and filters. For instance, accurate quartz-based electro-mechanical oscillators are required in many electronic circuits because of their extraordinary frequency selectivity, which can be orders of magnitude better than purely electronic RLC resonators. For this reason, for instance, typical consumer electronics may contain several electro-mechanical resonators for timing and frequency control.^25^ Though other types of electro-mechanical resonators could be used,^25−27^ quartz resonators are by far the most widely used because of their very high quality factors and high accuracy of the initial resonant frequency at low cost (down to fractions of a dollar). However, our approach can obviously be easily extended to other types of electro-mechanical resonators suitable for in-liquid operations. The control of the resonant frequency of quartz resonators can be easily achieved by feedback in case of high-vacuum techniques as both the deposition of the additional material on top of the electrodes^28^ and the partial removal of some electrode materials^29^ allow to tune the resonant frequency and, if the tuning is performed within an automatic feedback loop, the final resonant frequency can be controlled with errors within a few ppm.^30^ On the other hand, this type of tuning process has a very small range of frequency variation and can be used in a systematic way only in case of most common resonance frequencies (e.g., 32,768 Hz for watches). In fact, existing methods for manufacturing quartz with custom, non-standard resonant frequencies generally require the thermal evaporation of metals before packaging^31,32^ or only by adopting a much more complicated and expensive (e.g., around US $ 30 for a single device) quartz enclosure,^33−35^ after packaging. Moreover, the quality factor of the final resonator is generally extremely high, which can be an issue for the implementation of filters.

Here, we demonstrate the feedback-assisted solution growth of ZnO nanowires on the electrodes of conventional quartz crystals for enabling a simple, wet-chemical, low-cost post-fabrication adjustment of the resonant frequency, with tuning range (e.g. up to 1%) and spread (e.g. ±0.05%) which are comparable with more complex dry methods (e.g., 0.4% tuning range^36^ and ±0.05% spread^37^). Moreover, the proposed method also allows to effectively adjust, for a given final resonance, the quality factor of the resonator, which can be a key advantage for filters.

**Figure 1:**
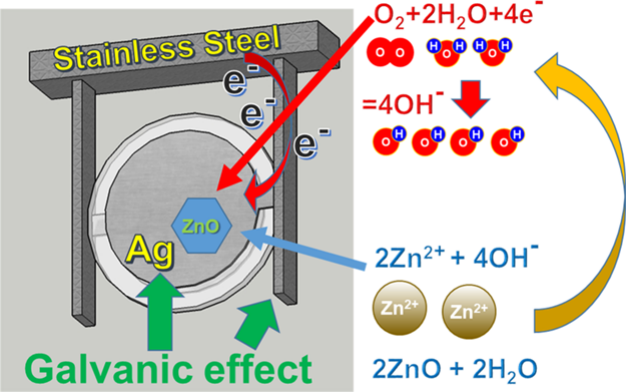
Schematic illustration of the proposed strategy for tuning electromechanical resonators by the solution-growth of ZnO nanowires; the tuning accuracy can be significantly improved by feedback (i.e.*,* real-time monitoring of the ZnO nanowire growth by means of a network analyzer).

**Figure 2:**
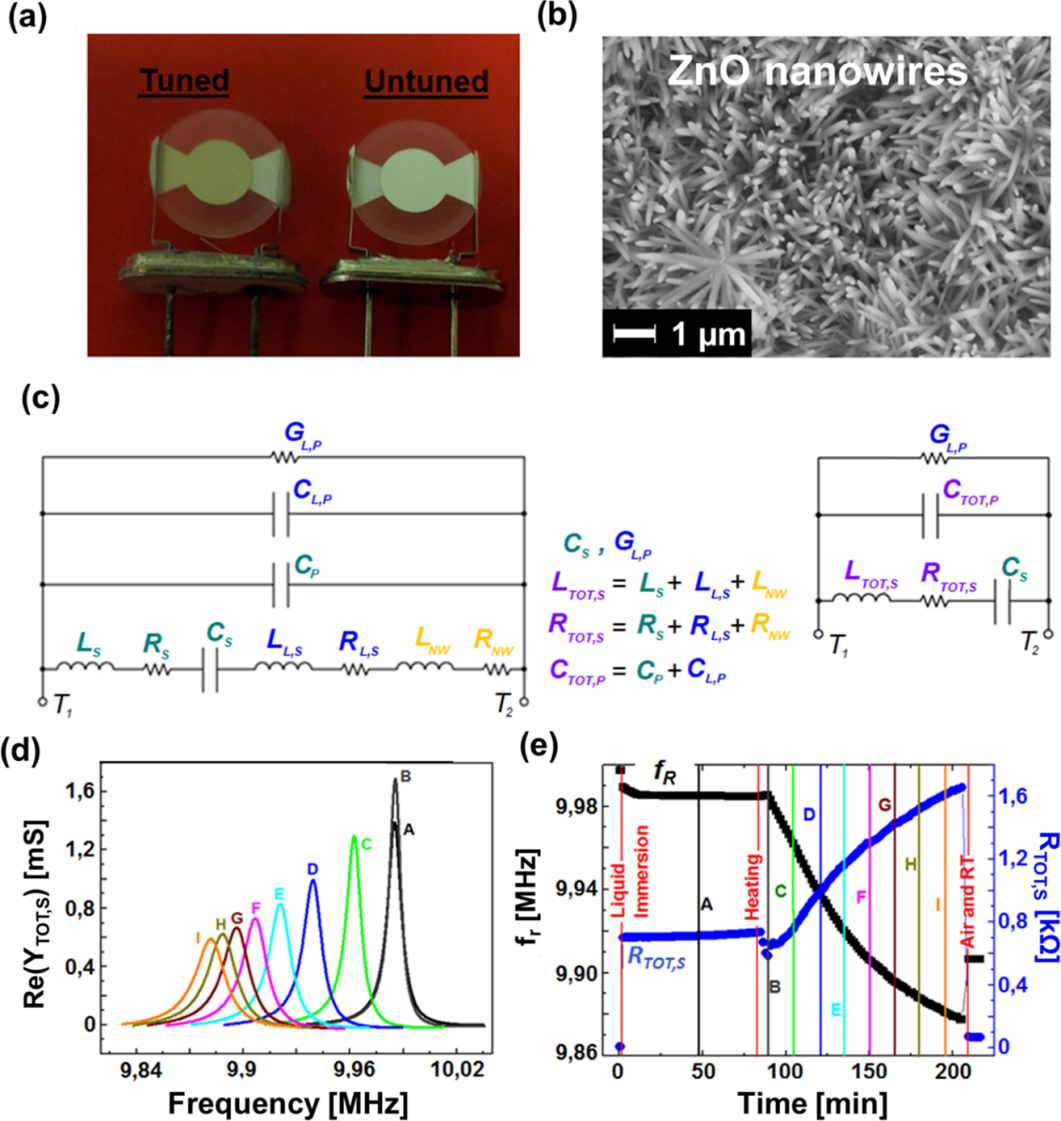
(a) Photo of an opened AT-cut quartz resonator (right element) and of another quartz resonator of the same type after the tuning procedure (left element). (b) Typical SEM image of ZnO nanowires grown on the silver electrodes of a quartz microbalance. (c) Equivalent electric circuit of the quartz in liquid (left) and simplified circuit (right). (d) Real part of the total admittance of the series RLC resonant circuit during the experiment (90 °C, 2.5 mM equimolar concentrations of zinc nitrate and hexamine). (e) Resonant frequency (black squares line) and total series resistance (blue dots line) as functions of time.

**Figure 3:**
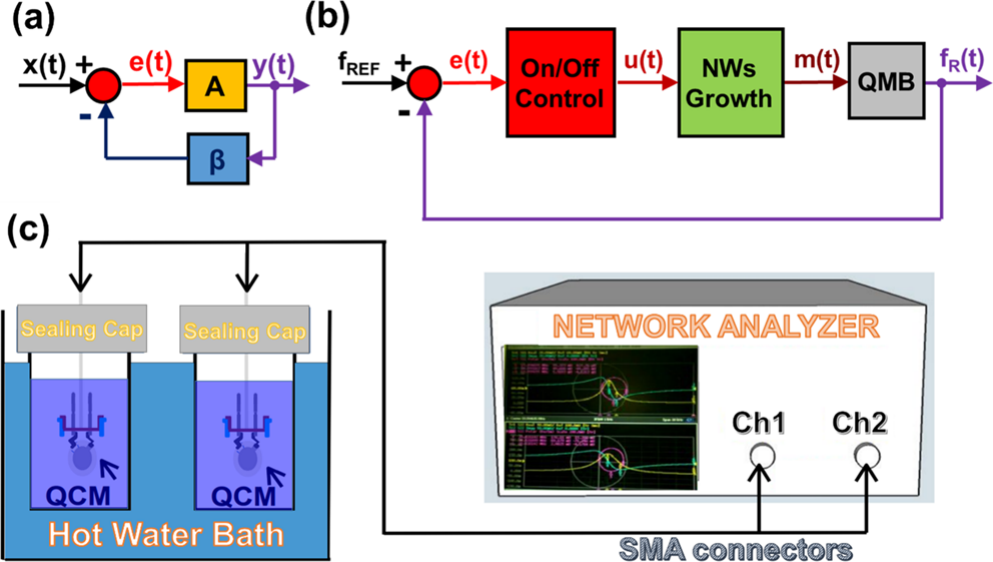
(a) Typical block model of a feedback system. (b) Block model of our feedback-assisted system for resonators tuning. (c) Experimental setup; the pyrex bottles containing the quartzes to be tuned (QCMs) are immersed in a thermostated water bath and the admittances of the QCMs are continuously monitored by the double channel network analyzer connected to the QCMs by BNC coaxial cables.

**Figure 4:**
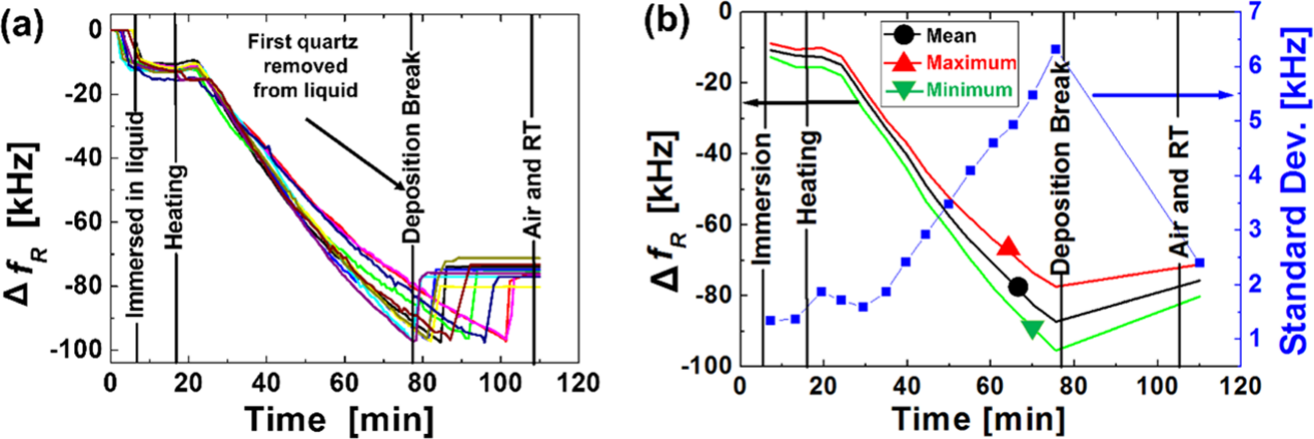
(a) Variation of the resonant frequency, Δ*f*_R_, as a function of time for 11 quartzes (growth at 90 °C in a 5 mM equimolar zinc nitrate hexahydrate and HMTA nutrient solution); all the quartzes were extracted from the solution and rinsed with a hot air stream immediately after the frequency of 9.9 MHz was reached. (b) Minimum (green), maximum (red), and mean (black) values of Δ*f*_R_ (left axis) and its standard deviation (blue line with dot squares, right axis).

**Figure 5:**
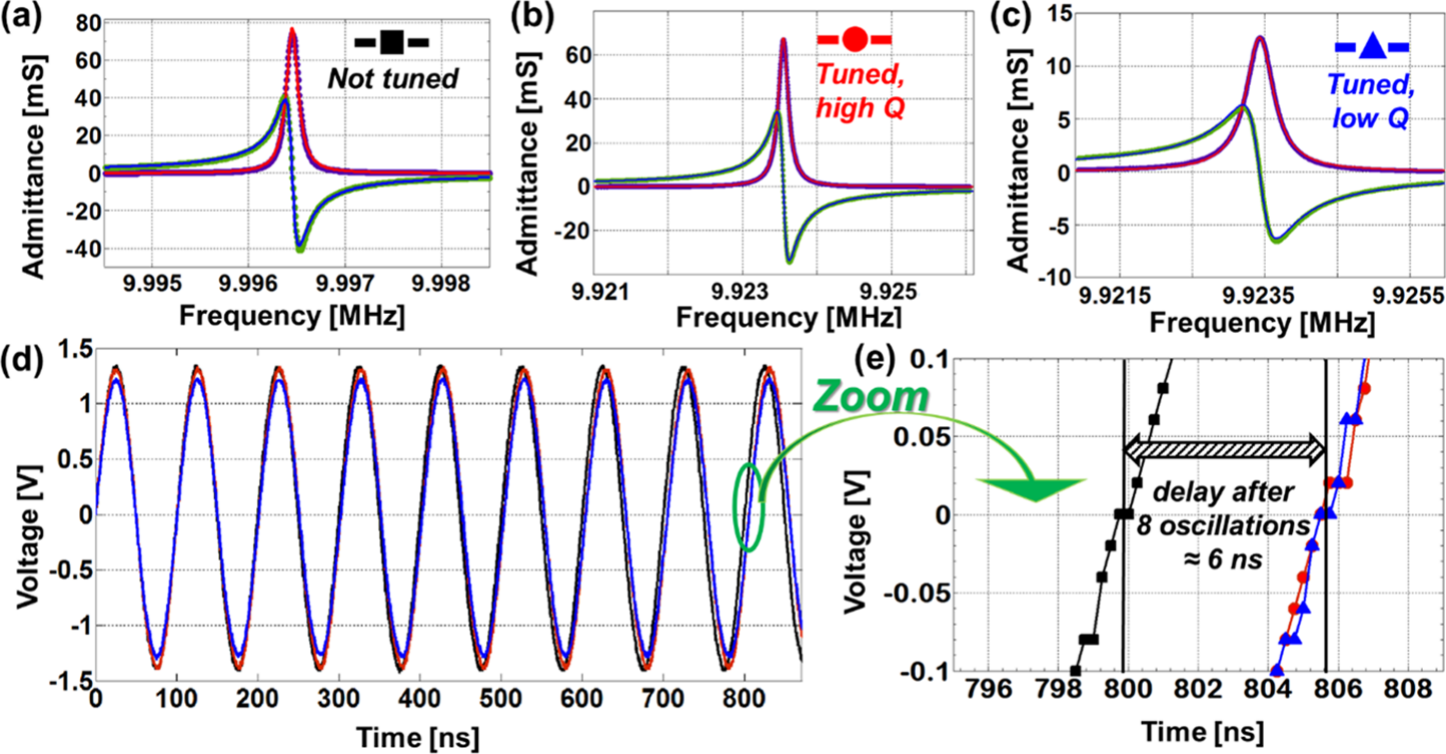
(a–c) Real part (red line with purple dots) and imaginary part (blue line with green dots) of the admittances of an untuned quartz (a), of a quartz after conventional deposition of ZnO nanowires (b), and of a quartz after slow deposition of ZnO nanowires (c). (d) Output voltage of an oscillator circuit driving the same untuned (black curve with filled squares), tuned (red curve with filled circles), and slowly tuned (blue curve with filled triangles) quartzes; for clarity, all the oscillations have been synchronized at *t* = 0 s. (e) Zoomed images of (d), showing the small difference between the two tuned resonant frequencies, independently on the nanowire growth rate.

The rest of this paper is organized as follows: after schematically illustrating the proposed strategy for tuning electromechanical resonators by the solution-growth of ZnO nanowires (Figure 1), we show the equivalent electric circuit of the quartz in liquid and how both the admittance of the series RLC resonant circuit and the resonant frequency change with time during the solution growth of ZnO nanowires on the quartz electrodes (Figure 2). Afterward, we discuss the use of feedback for tuning the quartz resonators by wet-chemical synthesis of ZnO nanowires (Figure 3) and show experimental results for a set of 11 quartzes (Figure 4). Finally, we compare an untuned quartz, a quartz after conventional deposition of ZnO nanowires, and a quartz after slow deposition of ZnO nanowires (Figure 5), thus illustrating the possibility to independently modify the resonant frequency and the quality factor of the quartz.

## Experimental Section

We used as electro-mechanical resonators commercial quartzes with silver electrodes and initial resonant frequencies equal to 10 MHz (Fox Electronics FOXLF100-20); for these type of quartzes the cap can be opened by cutting the metallic cover near the base (see^24^ for details). Afterward, we prepared an equimolar concentration of zinc nitrate and hexamine (reagents bought from Sigma-Aldrich) by mixing the two salts separately in DI water at room temperature (e.g., 0.3718 g of zinc nitrate and 0.1752 g of hexamine in 0.5 L of DI water, corresponding to equimolar concentrations of 2.5 mM). As a container for the growth, we used standard borosilicate glass bottles with 250 mL of volume, with a plastic cap modified to allow the insertion of a sliding Teflon bar with electrical feedthroughs at its end. This arrangement provides the electrical contacts to the resonator and allows to entirely immerge the quartz inside the chemical nutrient solution for allowing double-sided growth and therefore faster tuning of the resonator (i.e., the nanorods grow on both the electrodes of the quartz and, therefore, for a given growth period, the total mass change of the quartz is approximately doubled in comparison with single-sided growth). In order to start the nanostructure growth, we immersed the glass bottles containing the resonators to be tuned into a preheated thermostatic bain-marie at a temperature of 90 °C. We monitored the frequency of the quartz resonators by connecting with coaxial cables the two terminal contacts of the HC-49/U package with the two-channel Agilent E5070 network analyzer. In particular, we recorded the real and imaginary parts of the admittances by measuring the parameters S_11_ and S_22_. After the tuning, we tested the quartz resonators in a Colpitts circuit by measuring the oscillator output with the Agilent DSO-1002A oscilloscope. Finally, we tested the gain of the quartz filters as a function of frequency with the Bode 100 network analyzer by Omicron Lab.

## Results and Discussion

The solution growth of ZnO nanowires usually requires the pre-deposition of a seed layer on the substrate. However, there are methods such as, for instance, the spin-and-spray^38^ approach or ultra-efficient thermo-convective solution-growth,^39^ for directly growing ZnO nanowires without the issues associated to the seed layer deposition and, in several cases, the subsequent annealing. Similarly, as shown in Figure 1, recently, it has been found^40,41^ that the creation of the galvanic effect can promote the ZnO nucleation and, therefore, the growth of very dense arrays of nanowires on the metal that is not galvanically active (i.e., noble metals). Remarkably, this approach allows to easily grow high density ZnO nanowires on the silver electrodes of conventional low-cost quartz resonators by simply immersing the quartz in a conventional equimolar zinc nitrate-HMTA nutrient solution.^19,24^ With this method, ZnO nanowires can be grown already at temperatures above 65 °C (e.g. 70 °C^19^). Although the storage temperature range for the quartz crystal resonators used in our experiments was [−40, 85 °C], we set the temperature of the nutrient solution to 90 °C for consistency with previous experiments.^19,24^ Such a temperature, which is slightly higher than the maximum storage temperature (85 °C), is expected to be not critical because of the short duration of the nanowire growth (e.g. around 2 h) but may reduce the *Q* for longer periods (see later). As shown in Figure 1, this wet chemical strategy enables a straightforward tuning of the resonance properties of quartz resonators, with the additional advantage that the tuning accuracy can be easily improved by feedback (i.e., real-time monitoring of the ZnO nanowire growth by means of a network analyzer).

Figure 2a visually shows the difference between a standard QCM and a QCM after ZnO nanowires have been grown on its electrodes. The untuned resonator on the right side has a silver surface with a metallic grey color and a light reflecting behavior, whereas the tuned resonator on the left side has a milky color and is opaque, with an increased surface roughness due to the array of ZnO nanowires. Figure 2b shows a typical scanning electron microscopy (SEM) image of ZnO nanowires grown on the silver electrodes of quartz electro-mechanical resonators.^19,24^ The deposition of the array of ZnO nanowires over the electrodes of the QCM induces a variation Δ*f*_m_ of the quartz resonant frequency, for rigid and small variations of the mass loading, Δ*m*, according to the Sauerbrey equation^42^
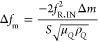
1where *f*_R,IN_ is the initial resonant frequency, *S* is the piezoelectric active area, and μ_*Q*_ and ρ_*Q*_ are the shear stiffness and the mass density of the quartz, respectively. The quartz can be modeled by the simple Butterworth-Van Dyke equivalent circuit, that is a series resonant RLC circuit with a parallel capacitance. However, since the quartz resonator is immersed into the conductive nutrient solution, the resonant frequency is also affected by the viscous coupling with the liquid, the surface roughness, surface stresses, and both the liquid conductance and the dielectric constant.^43,44^ In such conditions, starting from standard models,^45^ we derived the lumped equivalent (from the point of view external to the quartz terminals *T*_1_ and *T*_2_) circuit shown in Figure 2c, which extends the classical Butterworth-Van Dyke model (i.e. the capacitance *C*_P_ and the series RLC circuit constituted by *R*_S_, *L*_S_, and *C*_S_) by adding the liquid contribution (*L*_L,S_ and *R*_L,S_ for liquid loading, *G*_L,P_ for the liquid conductance, and *C*_L,P_ for the dielectric constant of the liquid) and the mass loading due to the nanostructure growth (*L*_NW_ and *R*_NW_). The total admittance *Y*_TOT,S_ of the series resonant RLC circuit can be expressed as
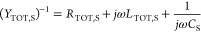
2

After the quartz admittance is measured, curve fitting allows to extract the five independent parameters of the equivalent electric circuit (the simplification to only five independent parameters is schematically shown in the right part of Figure 2c). The liquid conductance in parallel with the resonator (which could be removed by a slightly more complex arrangement with only one electrode exposed to the nutrient solution at the cost of a reduced tuning rate) adds losses due to the presence of *G*_L,P_ and, therefore, reduces the quality factor of the resonator (especially at high ionic concentrations). Nevertheless, our results demonstrate that feedback-assisted nanostructure growth is easily possible even with the simplest arrangement and in the presence of the additional losses introduced by *G*_L,P_. In order to extract the parameters of the resonator equivalent circuit, we measured the admittance of the resonator in a bandwidth around the resonant frequency by means of a network analyzer. As an example, Figure 2d shows the real part of *Y*_TOT,S_ (see eq 2) immediately after immersion of the quartz in the pyrex container with the nutrient solution (2 mM equimolar concentration of zinc nitrate hexahydrate and HMTA) at room temperature (A), after 15 min of immersion of the pyrex container in the thermal bath pre-heated at 90 °C (B), and at subsequent instants (C– I), separated by 15 min intervals. Figure 2e shows the resonant frequency *f*_R_ (black squares line) and the total series resistance *R*_TOT,S_ (blue dots line) as a function of time.

As expected, the resonant frequency decreases and the resonance peak widens with time due to gradual increase of the mass deposited on the microbalance and increased losses. The kinetics of the chemical growth of nanowires, related to the resonant frequency *f*_R_ of the total series RLC circuit, is accurately monitored by the network analyzer. Similarly, the increased electrode roughness can be monitored by means of the total series resistance *R*_TOT,S_. As evident from Figure 2d,e, after the changes due to both the immersion in liquid and the heating to the final growth temperature, there is a decay of the resonant frequency *f*_R_ due to the gradual growth of the ZnO nanowires.

There are no problems with self-heating of the quartz during measurements. In fact, not only the thermal resistance between the quartz immersed in the liquid nutrient solution and the environment is very small (much smaller than the typical thermal resistance between a similar quartz in air and the environment, which is typically around 300 K/W^46,47^), but the power required by the network analyzer for measuring the quartz impedance is also very small. For instance, in our experiments, we set the maximum power of the Agilent E5070 network analyzer to 1 mW, with measurements taking about 2 s and performed every 2 min, thus resulting in an average power of around 0.2 mW. Therefore, even with the quartz in air, such a small (0.2 mW) average power would result in an overheat of around 0.2 mW*300 K/W, that is 60 mK. In our case, the quartz is immersed in the nutrient solution and, therefore, the thermal resistance between the quartz and the environment is much smaller than 300 K/W, so that the quartz self-heating is even smaller than 60 mK and may be ignored. Consistently, in previous experiments on the solution growth of ZnO nanowires on the electrodes of quartz resonators, no artifact could be associated to the real-time monitoring of the resonant frequency.^19^

Tuning the resonant frequency of an electromechanical resonator corresponds to reducing the resonant frequency, in air and at room temperature, *f*_R,FIN_, to a desired value, lower than the initial resonant frequency, in air and at room temperature, *f*_R,IN_. Since *f*_R,FIN_ is not immediately available during the growth, it is useful to observe that
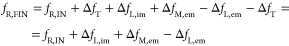
3where Δ*f*_T_, Δ*f*_L,im_, Δ*f*_M,em_, and Δ*f*_L,em_ are the variations of the series resonant frequency due to the difference between room temperature and the solution temperature during growth (Δ*f*_T_), the liquid loading immediately after immersion (Δ*f*_L,im_), the deposited mass immediately before the emersion (Δ*f*_M,em_), and the liquid loading immediately before emersion (Δ*f*_L,em_), respectively (note that, for simplicity, the variations, at the times of immersion and emersion, respectively, of the series resonant frequency due to the difference between room temperature and the solution temperature can be considered as identical and therefore simplified in eq 3). In principle, the series resonant frequency, in liquid and during growth, *f*_R,OL_, can be continuously on-line monitored and can be expressed as
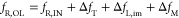
4where Δ*f*_M_ is the deposited mass which obviously changes with time. The difference between *f*_R,FIN_ and *f*_R,OL_ is therefore
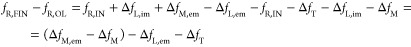
5where  is the difference between the deposited mass immediately after emersion and the deposited mass during on-line monitoring and, therefore, vanishes at the moment of emersion.

If we try to obtain a desired value for *f*_R,FIN_ (which corresponds to a desired value for the deposited mass) by on-line monitoring *f*_R,OL_, there are three sources of errors: Δ*f*_T_ which will, however, be usually negligible and can be taken into account or minimized by choosing proper quartz crystal cuts and calibration with preliminary experiments;  which can be somehow predicted if the procedure for interrupting the growth process is accurately defined and can be minimized if this procedure is sufficiently fast; Δ*f*_L,em_ which may not be accurately taken into account because though Δ*f*_L,im_ is obviously known during the synthesis (it is measured immediately after the immersion), Δ*f*_L,em_ is different from Δ*f*_L,im_ because these variations depend on the surface morphology which changes with the growth of the nanostructures and is affected by spread.^48^ In fact, in our experiments, the contributions of Δ*f*_L,em_ dominated the total error which, however, was still sufficiently low for enabling an effective feedback system for the tuning of the quartz resonator.

As shown in Figure 3a, in a feedback system, it is required to continuously measure the output *y*(*t*) during the experiment or the process to be controlled. The difference between the input *x* and β*y*, where β represents the feedback path, is the error signal *e*. Therefore, by using the real time measurement of the resonant frequency, *f*_R,OL_, obtained from the maximum of the conductance curve, we used feedback for tuning the resonator frequency by means of the ZnO nanowires grown on its silver electrodes (see Figures 3b and S1). The feedback loop compares the monitored output frequency of the quartz resonator with a reference frequency *f*_REF_ that can be calculated in real time by considering all the possible variations of the quartz resonant frequency within the process, as discussed before: . The subtraction between the two frequencies generates an error function *e*(*t*) that is used as an ON/OFF control for enabling/disabling the nanowire growth.
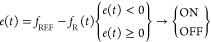
6

The nanostructure growth increases the mass *m*(*t*) deposited on the quartz electrodes and then reduces the resonant frequency of the resonator, which is a mass–frequency transducer. The ON/OFF control enables the movement of the quartz from a position in solution *p*(*t*) = LOW to a position outside the solution *p*(*t*) = HIGH, so that the nanostructure deposition is interrupted [*m*(*t*) = constant], as follows
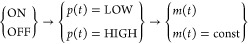
7

Figure 3c schematically shows the experimental setup composed of a hot water bath able to contain more Pyrex bottles, each one containing a Teflon bar with a fixed resonator to be tuned, respectively, connected to a channel of a network analyzer by coaxial cables and water-proof electrical feedthroughs. For simplicity, we used only one quartz in each Pyrex bottle with 250 mL of solution volume so that the independent interruption of the nanostructure growth was very simple but, in principle, more quartz resonators can be tuned in the same bottle, assuming, obviously, that the container cap allows to insert an independent Teflon bar for each resonator, with independent electrical contacts. In fact, even if it is possible to interrupt the growth by unscrewing the cap of the Pyrex bottles, Teflon bars sliding onto silicone O-rings can enable faster growth interruptions and, therefore, can reduce the frequency error given by Δ*f*_M,em_ – Δ*f*_M_, as discussed before. The system for removing the quartz from the nutrient solution could also be automatized by including a simple zeta-direction linear motor. For simplicity, we used a two-channel network analyzer for measuring the quartz admittances and therefore only tuned two resonators in each experiment, but clearly, oscillators for in-liquid operation can also be used.^49,50^ There are many commercially available, low-cost quartz resonators but only with a few well-defined resonant frequencies, and therefore, there are many frequency gaps which can be very large (e.g., from 9.84 to 10 MHz or even larger, from 10.24 to 11 MHz). As a proof of concept of the potential of our feedback-assisted wet chemical tuning, we fabricated quartz filters with frequencies in the middle of the gap between 9.84 and 10 MHz. In order to grow the ZnO nanostructures on the quartz electrodes, we immersed each quartz, with the initial resonant frequency in air equal to 10 MHz, in a 5 mM equimolar zinc nitrate hexahydrate and HMTA nutrient solution at 90 °C. For simplicity, the quartzes were extracted from the solution and rinsed with a hot air stream immediately after the in-liquid resonant frequency, measured in real-time by the network analyzer, was reduced below 9.9 MHz by the nanostructure mass. Figure 4a shows the variations of the resonant frequency, *Δf*_R_, as a function of time for 11 quartzes. Figure 4b shows the minimum (green), maximum (red), and mean (black) values of *Δf*_R_ and its standard deviation; at the deposition-break line, the quartz with the fastest growth rate is removed from the nutrient solution so that after this instant, its resonant frequency is measured in air. As expected,^19^ there is significant spread among the growth rates found for different, nominally identical quartzes kept in nominally identical (chemical concentrations and temperature) solutions. As a result, the target (in-liquid) resonant frequency is reached at significantly different time instants. Nevertheless, the online measurement of the resonant frequency, *f*_R_, which is the feedback signal, effectively enables the ultra-low-cost (e.g., no clean room or vacuum deposition techniques are required) tuning of the resonant frequency with a significantly lower final spread (about 2.4 kHz) in comparison with the correspondent open-loop process using a fixed deposition time (about 6.3 kHz). The proposed tuning procedure may find applications in sensors, oscillators, and filters. Additionally, the accurate tuning of the resonant frequency corresponds, for a given mass density, shear modulus, and active area of the quartz, to the accurate control of the deposited mass. Figure 4 only shows the real-time measurement of the quartz resonant frequency during the wet-chemistry process. However, the resonance series resistance *R*_TOT,S_ can also be found from the real part of the admittance spectrum. As shown in Figure 2d, the total series resistance is dramatically increased by the immersion in liquid (around 700 Ω initially and up to more than 1.5 kΩ, after the nanostructures growth) and is comparatively only slightly increased by the nanostructures after emersion (few tens of Ω), thus showing that the contribution of the liquid load *R*_L,S_ (which also depends on temperature and on the nanowires) largely exceeds the contribution of the ZnO nanowires in air, *R*_NW_, so that it would be very difficult to accurately control the final value of *R*_TOT,S_ in air by measurements taken with the immersed quartz during the growth of the nanowires.

The wet chemistry reaction also reduces the quality factor of the resonance and softens the frequency filtering characteristics of the device. In order to make more evident this phenomenon, we prepared a modified solution with an interfering agent for the ZnO nanowires synthesis. As a result, the mass growth on top of the electrodes is slowed down, thus increasing the time needed for reaching a desired resonant frequency and diminishing the final resonance quality factor. Specifically, we found that the solution contamination with a small amount of standard epoxy resin is a very simple way to slow down the ZnO chemical reactions. In practice, we added to the nutrient solution a small quantity of epoxy resin, namely about half a gram, which is close to the total weight of the main reagents (about 0.3718 g for zinc nitrate and about 0.1752 g for HMTA). As shown in Figure 5, we measured the quartz admittances of an untuned quartz and of two quartzes tuned with the fast (i.e. conventional nutrient solution) and with the slow growth (i.e. conventional nutrient solution with the addition of about half a gram of epoxy resin) procedures, respectively, and, subsequently, have connected the same quartzes to a conventional oscillator circuit. Figure 5a–c show the admittances around the resonant frequency of the different quartzes (tuned, slowly tuned and not tuned). The conductance is represented by blue dots with a fitting red line (always positive with a lorentzian peak shape), while susceptance is represented by green dots with a fitting blue line. Figure 5a shows the admittance of a standard not tuned commercial QCM (nominal resonant frequency of 10 MHz). Figure 5b shows the admittance of the quartz tuned with the standard nutrient solution, which therefore maintained a good quality factor. Finally, Figure 5c shows the admittance of the quartz tuned with a contaminated nutrient solution and, as expected, the peak is much more broadened than the peak in Figure 5b, relative to the quartz tuned with a standard nutrient solution. The Supporting Information Table S1 summarizes the fitting data relative to the BVD model of the three different quartz resonators. Remarkably, there is very small difference among the resonant frequencies of the two tuned quartzes, thus demonstrating the accuracy of the frequency tuning for both the tuning procedures, but there is a large spread of the series resistance *R*_S_ which is only slightly increased (from 12.9 to 14.75 Ω, Table S1) in the standard tuning process, but is more than doubled (from 15.6 to 39.5 Ω, Table S1) in the slow deposition. Since the series resistance determines both the peak bandwidth, Δ*f*, and the quality factor of the RLC series resonator, *Q* = *f*_R_/Δ*f* = 2π*f*_R_*L*_S_/*R*_S_, the peak bandwidth only slightly changes after the standard tuning (from 150 to 165 Hz, i.e., about 10% increase), but more than doubles (from 200 to 462 Hz) after the slow tuning. Since, for small changes of the resonant frequency, *Q* = *f*_R_/Δ*f* is approximately inversely proportional to the peak bandwidth, the *Q* is only slightly reduced (from about 68,600 to about 61,300, i.e., around 10% reduction) after standard tuning (which only slightly increase *R*_S_), but is more than halved (from above 52,100 to about 21,300, i.e., a reduction around 59%) after slow tuning (which more than doubles *R*_S_), as also evident from the significantly smoother peak of the real part of the admittances of the “low *Q*” tuned quartz (Figure 5c) in comparison with the correspondent and more sharp peaks of both the original (“not tuned”) quartz (Figure 5a) and of the “high *Q*” tuned quartz (Figure 5b). The possibility to independently tune the resonant frequency and the *Q* can be a crucial advantage for the implementation of electronic filters whose quality factor in many cases cannot be excessively high. Figure 5d,e demonstrate the effect of the tuning processes of Figure 5a–c in oscillator circuits. In practice, the three different quartz resonators (not tuned, black line with black squares; fast tuned, red line with red dots; slow tuned, blue line with blue triangles) have been included in conventional oscillator circuit, with an output voltage amplitude around 3 V. In order to graphically illustrate that our tuning method allows to design high-performance oscillator circuits with frequencies well within the frequency gap, Figure 5d shows the sinusoidal waveforms of the three oscillators. For clarity, we have synchronized all the waveforms at the origin of the graph (i.e. the phase of all the sinusoidal waveforms is zero at the instant *t* = 0). After a few cycles (eighth oscillations) the time delay between the fastest and the slowest waveforms is almost 6 ns (zoom in Figure 5e). Most remarkably, the accuracy of the frequency tuning method is such that both the oscillators comprising the fast-tuned and the slow-tuned quartz are still very well synchronized (Figure 5e). Clearly, the dependence of the resonating frequency on humidity, strain or temperature is undesired. However, after tuning, the quartz resonators can be easily re-assembled in their original packages and hermetically sealed, so that, during normal operations, the devices will not be subject to significant humidity or strain. As an example, in order to test the insensitivity of the final re-sealed devices to humidity, we re-assembled a tuned quartz in its original package which has then been taped and hermetically sealed by the epoxy resin. Afterward, we connected the tuned quartz (packaged and hermetically sealed) to an oscillator circuit (Figure S1), and for comparison we also connected a conventional quartz (without any tuning and with its original, hermetically sealed, package), used as a reference, to a similar oscillator circuit. In order to create an environment with high humidity (almost 80%), we poured warm water (pre-heated at about 40 °C) in a glass Petri dish capped by the plastic packaging of a humidity/temperature sensor. The LPC845 (Arm Cortex-M0+ based, low-cost 32-bit microcontroller with 30 MHz maximum operating frequency) from NXP was programmed in order to acquire the output voltage of the oscillator and to count the wave fronts (i.e., to measure the oscillating frequency) through the state configurable timer within a period of time settled by pulse-width modulation to 10 s. The frequency data were acquired and filtered with moving average over 30 samples. The quartzes were inserted in the almost completely closed air space between the water level and the plastic cap (see Figure S2). As evident from Figure S2, for both the tuned and the reference quartzes, very large variations of humidity did not result in significant changes of the resonant frequencies which only showed random fluctuations, thus confirming that the tuned quartzes can be easily re-assembled in a hermetically sealed package and then be practically insensitive to humidity. Clearly, unlike this work (post-fabrication tuning), the nano-tuning can take place before packaging the quartz resonator.

As to the dependence on temperature of the packaged quartz, for both a reference (no tuning) quartz and a tuned quartz, we connected each resonator to a conventional oscillator circuit (Figure S1) and then inserted both the quartzes in an oven from the top hole, so that the quartzes could be kept at a desired temperature (higher than room temperature). We tested both the quartzes at three different temperatures, namely, 50, 60, and 70 °C (i.e., the maximum nominal operating temperature of the FOX crystals used in this work). As shown in Figure S3, in both cases, we found comparable changes of the oscillating frequencies, which, as always, are determined by both the resonator (i.e., the quartz) and the oscillator circuit (Figure S1).

As an additional test, we also measured (Figure S4) the resonant frequency of a tuned quartz placed in the oven at a controlled temperature equal to 40 °C over a period of 10 h and found that the frequency changes are less than ±300 Hz, which for a resonant frequency close to 10 MHz, corresponds to approximately ±30 ppm, which are inside the frequency tolerance standards of the FOX crystal alone (the frequency stability is obviously also reduced by the non-idealities of the oscillator circuit).

## Conclusions

In summary, we successfully controlled the resonant frequencies of piezoelectric quartz resonators by depositing ZnO nanostructures on the quartz electrodes with low cost, low temperature wet-chemistry techniques. Moreover, instead of adopting an open-loop strategy with fixed deposition time, we used real-time measurements of the quartz admittance spectrum during the nanostructure growth in order to close the feedback loop and to more accurately tune the resonant frequency. This is the first report of functional nanowire-tuned oscillators and filters fabricated by feedback-assisted wet-chemistry synthesis of nanostructures. For validation, the tuned quartz resonators have been tested with standard electronic oscillator circuits. Our results confirm that accurate tuning of the resonant frequency is possible with a high tuning range (around 1% in our experiments). We also demonstrated that simple modifications of the nutrient solution and therefore of the reaction kinetics affect the quality factors of the quartzes, thus enabling the simple control, in addition to the resonant frequency, of the resonator quality factors. This result is of special interest for electronic filters whose bandwidth cannot be too narrow as it is typically the case of quartz resonators. The nanometer dimensions of the ZnO nanowires result in the accurate tuning of the resonant frequency, while the much larger (e.g., few millimeters) diameter of the quartz allows straightforward interfacing, simple handling, and packaging. Interestingly, ZnO is also widely employed for surface acoustic wave devices, so that our approach could be easily extended to this important class of electromechanical resonators.^51,52^ Moreover, the proposed approach can obviously be extended to other methods for growing ZnO nanowires^53−55^ or other quasi-1D nanostructures or composites.^56^

## Supporting Information Available

The Supporting Information is available free of charge. Parameters of the Butterworth-Van Dyke model of the electro-mechanical resonators extracted from the admittance spectra shown in Figure 5. Oscillator circuit. Experiments for verifying robustness against humidity and temperature. Long-term stability. (PDF)
