# Supplementary material for: ZnO Nanowires for Feedback-Assisted Tuning of Electromechanical Resonators
Source: ACS Appl Nano Mater. 2022 Sep 28;5(10):15817–25. doi: 10.1021/acsanm.2c03963 (PMC9623547; doi:10.1021/acsanm.2c03963)
Supplement: Supplementary file 1 — an2c03963_si_001.pdf [file an2c03963_si_001.pdf]

Supporting information

ZnO Nanowires for Feedback-Assisted Tuning of Electromechanical Resonators

Andrea Orsini[1,2], *Christian Falconi*[1]*

[1] Department of Electronic Engineering, University of Rome Tor Vergata, Via del Politecnico 1, 00133 Rome, Italy

[2] Facoltà di Ingegneria, Università degli Studi Niccolò Cusano, Via Don Carlo Gnocchi 3, 00166 Rome, Italy

* Correspondence to: falconi@eln.uniroma2.it

Parameters of the Butterworth-Van Dyke model of the electro-mechanical resonator

Table S1 shows the parameters of the Butterworth Van-Dyke model of the electro-mechanical resonator extracted from the admittance spectra shown in Figure 4(a-c). For validation, each relevant parameter (*i.e.* the resonant frequency, the series resistance, the series inductance, and the series capacitance) has been determined by best fitting both the real part of the admittance and the imaginary part of the admittance (values tagged by (Re) and by (Im), respectively); as evident, there is excellent agreement between the (Re) and the (Im) values.

Table S1. BVD parameters found from the admittance spectra in Figure 4(a-c).

BVD parameters	**Fast QCM (in)**	**Fast QCM (end)**	**Slow QCM (in)**	**Slow QCM (end)**
Resonant Frequency (f_R) (Hz)	9.996.453 (Re)<br>9.996.453 (Im)	9.923.545 (Re)<br>9.923.545 (Im)	9.996.205 (Re)<br>9.996.205 (Im)	9.923.428 (Re)<br>9.923.427 (Im)
Series Resistance (Ω)	12.91 (Re)<br>12.92 (Im)	14.76 (Re)<br>14.75 (Im)	15.66 (Re)<br>15.67 (Im)	39.5 (Re)<br>39.53 (Im)
Series Inductance (mH)	14.08 (Re)<br>14.17 (Im)	14.48 (Re)<br>14.50 (Im)	13.01 (Re)<br>13.06 (Im)	13.53 (Re)<br>13.53 (Im)
Series Capacitance (fF)	18.00 (Re)<br>17.89 (Im)	17.76 (Re)<br>17.74 (Im)	19.48 (Re)<br>19.41 (Im)	19.01 (Re)<br>19.01 (Im)

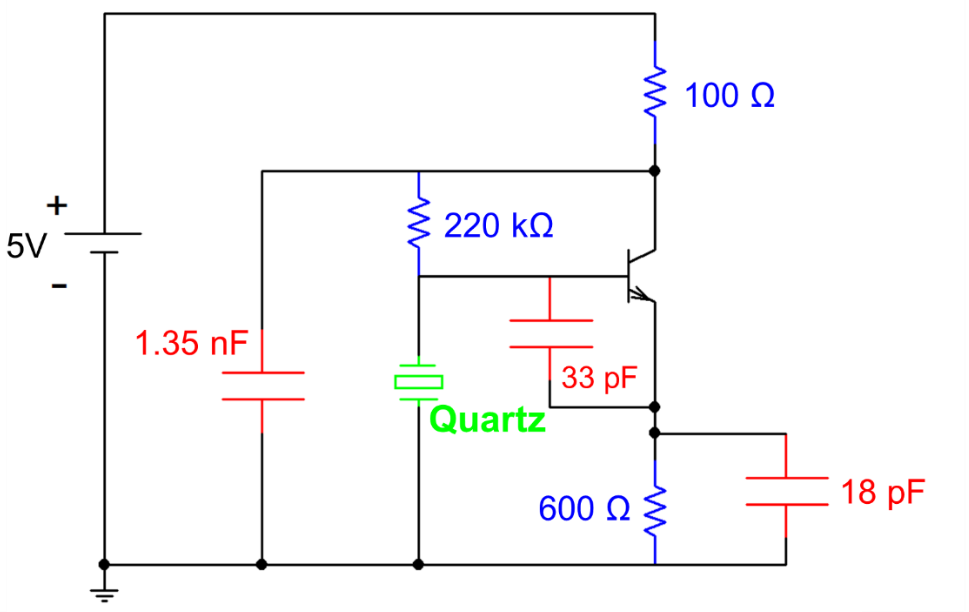

Figure S1. Oscillator circuit for driving the quartz resonators.

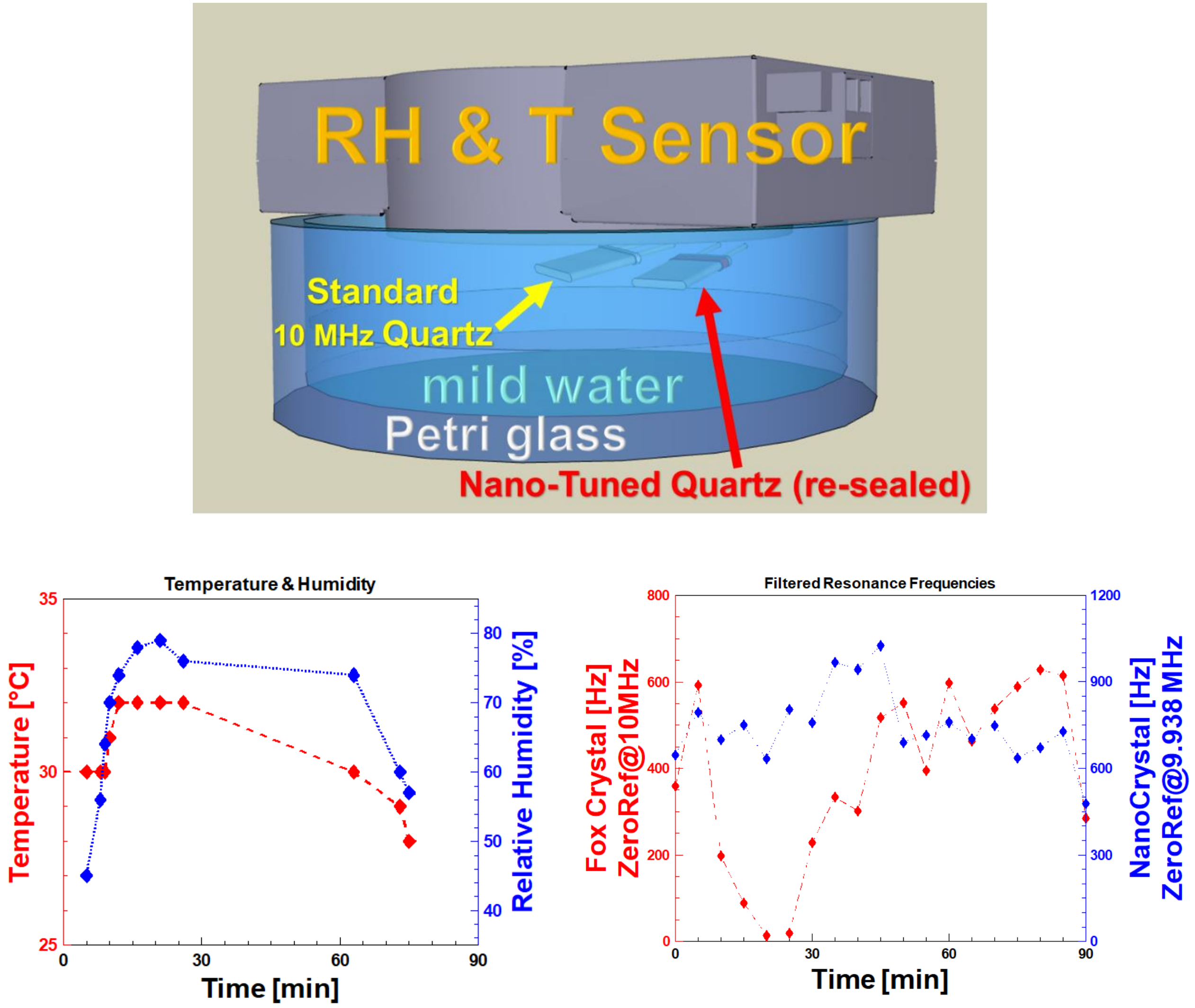

Figure S2. Experiments for verifying the insensitivity to humidity of the tuned quartz after re-packaging and hermetical sealing with epoxy. Experimental setup showing the reference quartz and the nano-tuned quartz exposed to high humidity by placing them within an almost closed Petri dish containing warm water, with a relative humidity and a temperature sensor (top figure). Time-evolution of the temperature and relative humidity measured by the sensors (bottom left figure) and of the correspondent oscillating frequencies (bottom right figure) for both the quartzes, each connected to a driving oscillator circuit.

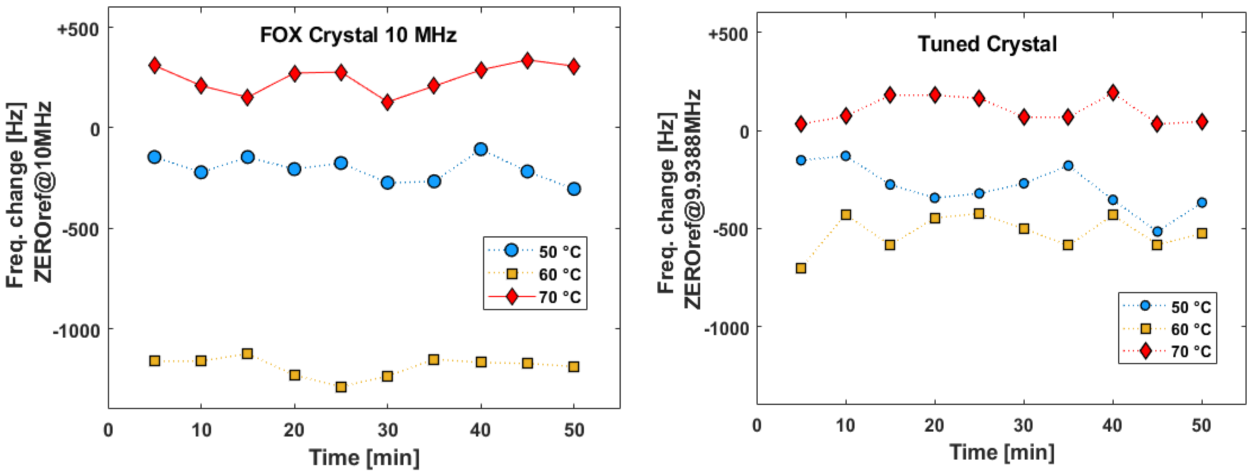

Figure S3. Time evolution of the oscillating frequencies for both a standard 10 MHz quartz (left figure) and for a nano-tuned quartz (right figure), each connected to a driving oscillator circuit.

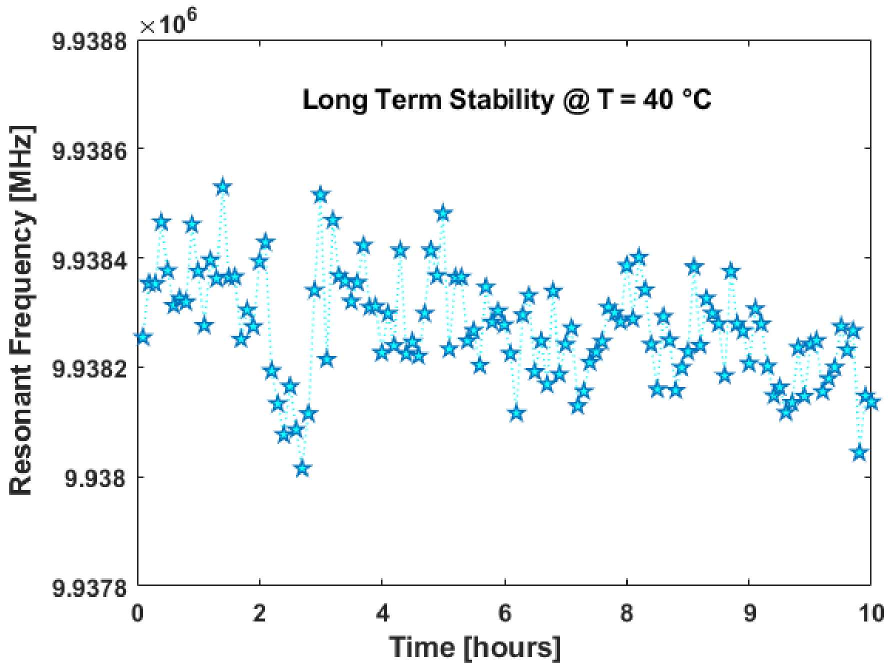

Figure S4. Long-term (10 hours) time evolution of the oscillating frequency for a nano-tuned quartz connected to a driving oscillator circuit.
